# Serum developmental endothelial locus-1 is associated with severity of sepsis in animals and humans

**DOI:** 10.1038/s41598-019-49564-5

**Published:** 2019-09-10

**Authors:** Won-Young Kim, Seung-Hwan Lee, Dong-Young Kim, Hyun Jin Ryu, Gyu Rak Chon, Yun Young Park, Yan Fu, Jin Won Huh, Chae-Man Lim, Younsuck Koh, Eun Young Choi, Sang-Bum Hong

**Affiliations:** 1 Department of Internal Medicine, Chung-Ang University Hospital, Chung-Ang University College of Medicine, Seoul, Republic of Korea; 2 Department of Biomedical Sciences, University of Ulsan College of Medicine, Seoul, Republic of Korea; 3 Department of Internal Medicine, Cheongju St. Mary’s Hospital, Cheongju, Republic of Korea; 4 Department of Pulmonary and Critical Care Medicine, Asan Medical Center, University of Ulsan College of Medicine, Seoul, Republic of Korea

**Keywords:** Diagnostic markers, Prognostic markers, Translational research, Sepsis

## Abstract

Disruption of the endothelial glycocalyx has a prominent role in the pathophysiology of sepsis. Developmental endothelial locus-1 (Del-1) is an endothelial-derived anti-inflammatory factor. We hypothesized that degradation of the endothelial glycocalyx during sepsis may increase serum Del-1. A mouse model of sepsis was created using cecal ligation and puncture. In septic mice, the endothelial glycocalyx was nearly completely degraded, with less formation of Del-1 in the endothelium and extracellular matrix than in control mice. Serum Del-1 levels were significantly increased in the septic mice with increasing severity of sepsis. Serum Del-1 levels were also measured in 84 patients with sepsis and septic shock and in 20 control subjects. The median serum Del-1 level in patients with sepsis was significantly higher than that in healthy controls. The high Del-1 group had higher illness severity scores and contained more patients with organ dysfunction than the low Del-1 group. The 90-day mortality rate was significantly higher in the high Del-1 group than in the low Del-1 group. Multivariate analysis indicated a tendency for a high serum Del-1 level to be associated with a higher mortality risk. Increased serum Del-1 may be a novel diagnostic biomarker of sepsis and an indicator of disease severity.

## Introduction

Sepsis is defined as life-threatening organ dysfunction caused by a dysregulated host response to infection^1^. Mortality in patients with sepsis has been attributed to endothelial dysfunction caused by inflammation^2^. Pro-inflammatory mediators disrupt the glycocalyx^3^ and changes in the structure of the glycocalyx and integrity of the vasculature result in capillary leak, microvascular thrombosis, systemic hypotension, and tissue hypoperfusion. Moreover, hypoxia in the tissues produces large quantities of reactive oxygen and nitrogen species^4^. These effects lead to endothelial dysfunction, refractory vasodilation, and disseminated intravascular coagulation (DIC).

Uncontrolled recruitment of leukocytes to sites of inflammation also contributes to fatal organ dysfunction during the course of sepsis^5^. Firm adhesion of leukocytes to the endothelium and subsequent transendothelial migration are mediated by interactions between leukocyte integrins and their endothelial counter-receptors^6,7^. Developmental endothelial locus-1 (Del-1) is an endogenous inhibitor of endothelial adhesion of leukocytes. Specifically, it antagonizes the interaction between lymphocyte function antigen (LFA)-1 and intercellular adhesion molecule (ICAM)-1^8^ as well as the binding of macrophage (Mac)-1 with complement fragment iC3b^9^. Del-1-deficient mice showed increased infiltration of neutrophils in acute lung inflammation^8^ and in the periodontal tissue causing spontaneous periodontitis^10^. On the contrary, locally administered Del-1 blocked inflammatory bone loss in nonhuman primates^11^.

Therefore, Del-1 may be an important homeostatic factor for preventing an inflammatory response in the endothelium and subsequent endothelial dysfunction^12^. Given that Del-1 is deposited in the endothelium and extracellular matrix^13^ and that degradation of the endothelial glycocalyx is common in the course of sepsis^3,4^, we hypothesized that circulating Del-1 may be increased with increasing severity of sepsis. If so, serum Del-1 could be a useful biomarker of sepsis and dysfunction in other organs.

In this study, serum Del-1 levels were examined in an animal model of sepsis and in humans. The relationship between the serum Del-1 level and the severity and prognosis of sepsis was the main focus of the investigation. Sepsis is presented with either direct injury to the lung characterized by more severe lung epithelial damage (pulmonary sepsis) or systemic inflammatory response characterized by more systemic endothelial injury (non-pulmonary sepsis)^14^. To assess expression of serum Del-1 over time in both models, serum Del-1 levels were measured in mice that underwent cecal ligation and puncture (CLP) and in mice with lipopolysaccharide (LPS)-induced pneumonia.

## Materials and Methods

### Mouse sepsis model

A schematic flow chart of the study can be found as Supplementary Fig. S1. The CLP procedure was conducted according to the methods described previously^15^. Healthy 6–8-week-old male C57BL/6 mice (22 g) were used. The mice were anesthetized with 3%–4% isoflurane with O_2_ flow at 2 l/min and restrained in the supine position. A 1-cm midline incision was made into the skin only; another 1-cm midline cut was then made into the peritoneum. After exteriorizing the cecum, the cecum was ligated at a point approximately 2 cm from the cecal tip with a 2-0 silk suture. A 21-gauge needle was used to puncture the cecum twice. In the experiment assessing Del-1 levels according to severity of sepsis induced by varying the size of the enterotomy, the cecum was also punctured twice with a 23-gauge or 25-gauge needle. The ligated and punctured cecum was replaced back into the abdomen and the incision closed. Next, 1 ml of a saline/buprenorphine mixture was injected into the peritoneal cavity.

For the LPS-induced sepsis model, healthy 6–8-week-old male C57BL/6 mice were used. The mice were anesthetized by intraperitoneal injection of 0.5 ml tribromoethanol (Avertin; Sigma-Aldrich, St. Louis, MO). A 1-cm midline incision was made into the skin only; when the trachea was exposed, another small incision was made just distal to the larynx. LPS serotype O111:B4 (catalog no: L2630; Sigma-Aldrich) dissolved in 40 μl of sterile 0.9% NaCl was instilled intratracheally via a 23-gauge syringe, followed by 0.15 ml of air. The dose of LPS used was 20 μg/mouse. After intratracheal treatment, the mice were kept in an upright position for 10 min to allow the fluid to spread throughout the lungs.

C57BL/6 mice (C57BL/6NCrljOri) were purchased from Orient Bio (Seongnam, Gyeonggi Province, Korea). Del-1^−/−^ mice on the C57BL/6 background were kindly provided by Prof. T. Chavakis (Dresden University, Germany)^8^.

### Solid-phase binding assay

Binding of Del-1 to immobilized heparan sulfate and syndecan-1 was tested on MaxiSorp 96-well plates (Nunc A/S, Roskilde, Denmark). The plates were coated with 50 nM heparan sulfate (Sigma-Aldrich), recombinant mouse syndecan-1 (R&D Systems, Minneapolis, MN), or bovine serum albumin (BSA) in phosphate-buffered saline (PBS) at 37 °C for 2 h. After washing with 0.05% PBS-Tween-20 (PBST), the plates were blocked with wash buffer containing 0.5% BSA at room temperature (RT) for 1 h. The plates were then incubated with 50 nM recombinant human Del-1 (R&D Systems) in PBS at 37 °C for 2 h. To test whether heparinase III (Sigma-Aldrich) inhibits Del-1-heparan sulfate or Del-1-syndecan-1 binding, the immobilized heparan sulfate or syndecan-1 was incubated with Del-1 (50 nM) for 2 h at 37 °C, and then, heparinase III was added at 0.005 U/ml or 0.05 U/ml. After 1.5 h, the plates were washed three times with wash buffer and incubated with 100 μl of Del-1 antibody (catalog no: 12580-1-AP; Proteintech, Rosemont, IL) (1:500 dilution in wash buffer) at RT for 1.5 h. After washing three times with wash buffer, 100 μl of anti-rabbit IgG-horseradish peroxidase (HRP) (Jackson ImmuneResearch, West Grove, PA) (1:1000 dilution in wash buffer) was added and the plates were incubated at RT for 1 h. Finally, the plates were washed three times with wash buffer and incubated at RT for 0.5–1 h with 100 μl of tetramethylbenzidine (TMB) solution (BD Biosciences, San Diego, CA). The absorbance at 630 nm was measured on a Synergy HT Microplate Reader (Biotek Instruments, Winooski, VT).

### Immunohistochemistry

The mice were anesthetized by intraperitoneal injection of 0.5 ml of Avertin and then perfused with PBS and 4% paraformaldehyde. Their lungs were then removed and fixed in 4% paraformaldehyde for 2 h, followed by sequential incubation in 15% and 30% sucrose. The tissues were then embedded in Optimal Cutting Temperature compound (Siegen) and frozen at −80 °C, and 15 μm cryostat sections were prepared. The sections were washed three times with PBS for 5 min, and permeabilized for 15 min with 0.1% Triton X-100 (Sigma-Aldrich). The sections were then incubated overnight at 4 °C with primary antibodies (1:100) against Del-1 (AbFrontier, Seoul, Korea), heparan sulfate (Amsbio, Abingdon, UK), E-cadherin (Cell Signaling, Danvers, MA), and CD31 (eBioscience, San Diego, CA), followed by secondary antibodies at RT for 1 h. The glycocalyx was stained with isolectin-fluorescein isothiocyanate (1:100; Sigma-Aldrich) for 1 h.

The inhibition of Del-1-glycocalyx binding by heparinase or phosphatidylinositol-specific phospholipase C (PI-PLC) was tested using immunohistochemistry. PI-PLC, released in abundance by some strains of *Staphylococcus aureus* and certain other bacteria, has been utilized experimentally to identify a class of proteins that are uniquely anchored to cell membranes by a glycan-phosphatidylinositol (GPI) moiety^16^. HEK293T cells or HEK293T cells expressing mouse Del-1 gene were cultured in 500 μl of DMEM-10 at 5 × 10^4^ cells per well in a cell culture plate containing gelatin-coated coverslips at 37 °C overnight. The cells were treated with heparinase (0.05 U/ml) or PI-PLC (2 U/ml) at 37 °C for 1.5 h and fixed with PBS containing 2% paraformaldehyde for 10 min at RT. After washing with PBS, the cells were treated with PBS containing 5% normal goat serum (Hyclone Labs, Ogden, UT) and 1% BSA (Sigma-Aldrich) for 1 h at RT. The cells were then incubated overnight at 4 °C with anti-mouse Del-1 antibody (1:250; AbFrontier, Seoul, Korea), followed by secondary antibody Alexa 488-conjugated goat anti-rabbit IgG (1:500; Invitrogen, Carlsbad, CA) at RT for 1 h. Nucleuses were stained with DAPI (2 μM; Invitrogen). After washing in PBS three times for 10 min each, the coverslips were mounted with Fluoromount-G (Electron Microscopy Science, Hatfield, PA). Images were captured using a laser-scanning confocal microscope (LSM 710; Zeiss Microscopy, Jena, Germany).

### Quantitative real-time polymerase chain reaction (RT-PCR)

Del-1 expression in various septic mouse tissues was assessed using quantitative RT-PCR. Total RNA was extracted from tissues at various sites in the septic mice using QIAzol reagent (Qiagen, Hilden, Germany), and cDNA was synthesized using the High-Capacity cDNA Archive kit (Applied Biosystems, Foster City, CA). The cDNA was amplified using LightCycler 480 SYBR-Green I Master and a LightCycler 480 machine (Roche, Mannheim, Germany). The PCR conditions were as follows: 95 °C for 15 min; 50 cycles of 20 s at 95 °C, 20 s at 60 °C, and 20 s at 72 °C, and 95 °C for 15 min. Melting curve analyses were performed to ensure that specific PCR products were generated. The data were analyzed using the comparative threshold (C_T_) method^17^, and the mRNA levels were normalized to those of 18 S RNA. The primers used were as follows: Del-1, forward: 5′-CCTGTGAGATAAGCGAAG-3′ and reverse: 5′-GAGCTCGGTGAGTAGATG-3′; 18 S, forward: 5′-CGCGGTTCTATTTTGGT-3′ and reverse: 5′-AGTCGGCATCGTTTATGGTC-3′.

### Enzyme-linked immunosorbent assay (ELISA)

To test whether heparinase or PI-PLC inhibits Del-1-glycocalyx binding, the Del-1 concentration in HEK293T cells or HEK293T cells expressing Del-1 was measured by ELISA. The cells seeded in a gelatin-coated 24-well plate at 3 × 10^5^ cells/well were incubated for 36 h and washed with warm PBS. The media was replaced with fresh media, the cells were incubated in the presence of heparinase (0.5, 2 U/ml) or PI-PLC (0.2, 2 U/ml) at 37 °C for 1 h, and the supernatants were collected and centrifuged. One hundred microliters of supernatants were added to MaxiSorp 96-well plates and incubated at 4 °C overnight.

To measure Del-1 concentrations in mouse and human, a MaxiSorp 96-well plate was coated with 50 μl of 200 ng/ml L-α-phosphatidylserine (Avanti Polar Lipids, Alabaster, AL) at 4 °C for 12 h^18^. After washing with 0.05% PBST three times, diluted samples were added, and the plate was incubated at RT for 3 h. Serial dilutions of recombinant human Del-1 protein (R&D Systems) were added as the standard. The plate was then washed, incubated with a rabbit anti-Del-1 antibody (catalog no: 12580-1-AP; Proteintech) at RT for 2 h, washed four times with PBST, and incubated with the HRP-conjugated anti-rabbit IgG (Jackson ImmuneResearch) at RT for 1 h. After five washes with PBST, the plate was incubated with TMB solution (BD Biosciences). Absorbance at 650 nm was read on a Synergy HT Microplate Reader (BioTek Instruments). To measure other biomarkers in mice, the capture antibodies were diluted in PBS and coated on a Maxisorp plate, and then incubated at 4 °C for 12 h. In some ELISA, serum was diluted and added to a Maxisorp plate that was not coated with capture antibodies, and then analyzed using detection antibodies. The antibodies used for mouse serum were as follows: anti-receptor for advanced glycation end products (RAGE) (catalog no: MAB11795; R&D Systems), anti-ICAM-1 (catalog no: 116113; Biolegend, San Diego, CA), anti-syndecan-1 (catalog no: 142503; Biolegend), anti-interleukin (IL)-6 (catalog no: 14-7061-68; eBioscience), anti-IL-17 (catalog no: 14-7175-68B; eBioscience), and anti-tumor necrosis factor (TNF)-α (catalog no: 14-7423-68 A; eBioscience). After three washes in 0.1% PBST, the plate was blocked with PBST containing 0.3% skim milk for 1 h at RT. The plate was then washed five times with 0.1% PBST, incubated with samples and the standard at 4 °C for 12 h, washed again, and incubated with the respective detection antibodies and HRP-conjugated secondary antibodies (Cell Signaling) at RT for 1 h. After five washes, the plate was incubated with TMB solution and the reaction was stopped with 1 N HCl. Absorbance was read at 450 nm on a microplate reader (BioTek Instruments). A Magnetic Luminex Performance Assay (catalog no: FCST03; R&D Systems) was used for quantification of IL-1β, TNF-α, and IL-6 in the human blood.

### Evaluation of infiltrating inflammatory cells

Del-1 regulation of inflammatory cell recruitment was tested in mice with CLP-induced sepsis. After 4 h of CLP (21-gauge), the mice were sacrificed and the blood, bronchoalveolar lavage (BAL) fluid, and peritoneal lavage fluid were collected. To evaluate infiltrating neutrophils, the cells were counted and preincubated with Mouse BD Fc Block purified anti-mouse CD16/CD32 mAb 2.4G2. The cells were then stained with specific antibodies phycoerytherin-conjugated anti-Gr1 (clone RB6-8C5; BD Biosciences) and allophycocyanin-conjugated anti-CD11b (clone M1/70; BD Biosciences) and analyzed by flow cytometry on a BD Accuri C6 instrument (BD Biosciences).

### Study subjects

Eighty-four patients diagnosed with sepsis or septic shock between March 2011 and January 2013 were enrolled. All patients were older than 18 years of age and had been admitted to the medical intensive care unit (ICU) of a university-affiliated tertiary care hospital in Seoul, Korea. Blood samples were collected after informed written consent was obtained from all patients and subject or their next of kin within the first day of admission. Twenty healthy individuals who had voluntarily undergone a private health examination at the same hospital in April 2014 were recruited as controls.

The baseline demographic and clinical characteristics that were collected were age, sex, comorbidities based on the Charlson Comorbidity Index^19^, source of sepsis, presence of bacteremia, septic shock, acute respiratory distress syndrome (ARDS), and/or DIC, and the status of the patient within 24 h of admission to the ICU, namely, whether the patient was being treated with mechanical ventilation and vasopressors. In addition, the severity of illness at the time of ICU admission was recorded; this was assessed using the APACHE (Acute Physiology and Chronic Health Evaluation) II score^20^ and the SOFA (Sequential Organ Failure Assessment) score^21^. Laboratory data included a complete blood cell count, coagulation profile, C-reactive protein, procalcitonin, and serum lactate. Overt DIC was defined according to the criteria of the International Society on Thrombosis and Haemostasis^22^. Sepsis and septic shock were defined using the Third International Consensus Definitions for Sepsis and Septic Shock (Sepsis-3)^1^. ARDS was diagnosed by a consensus definition^23^.

### Statistical analysis

Continuous variables are presented as mean ± SEM, as mean ± SD, or median and interquartile range. Categorical variables are presented as percentages. The two groups were compared in terms of continuous variables using the Mann-Whitney *U* or Student’s *t*-test, and in terms of categorical variables using the chi-square or Fisher’s exact tests. The optimal cutoff value for serum Del-1 predicting 90-day mortality was identified by receiver-operating characteristic (ROC) curve analysis. Kaplan-Meier estimates were built to predict mortality, and the curves were compared using the log-rank test. A Cox proportional hazards regression model using the forward conditional method was used to identify factors associated with time to 90-day mortality in the study patients. The areas under the ROC curves (AUCs) for the serum Del-1, APACHE II score, SOFA score, white cell count, C-reactive protein, procalcitonin, and serum lactate predicting 28-day and 90-day mortality were calculated. All analyses were performed using SPSS version 22.0 for Windows (IBM, Armonk, NY). All tests of significance were two-tailed. *P*-values < 0.05 were considered statistically significant.

### Ethical approval

The animal studies were approved by the Asan Institute for Life Sciences Institutional Animal Care and Use Committee (IACUC No. 2017-12-105). All animal experiments were performed in accordance with relevant guidelines/regulations. The human study protocol was approved by the Institutional Review Board of Asan Medical Center (2011-0643) and conducted in accordance with the amended Declaration of Helsinki.

## Results

### Endothelium and Del-1 in sepsis

**Figure 1:**
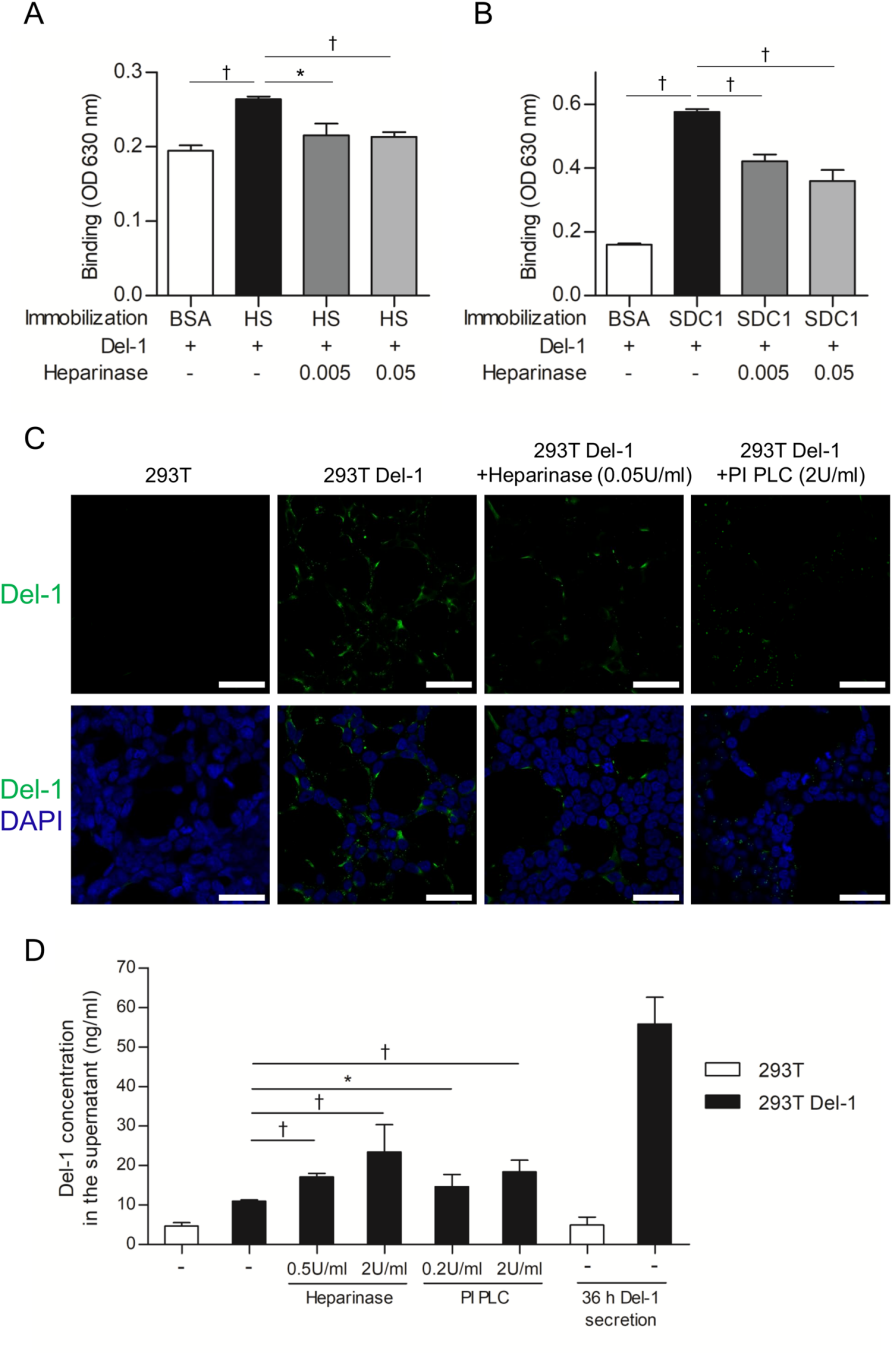
Del-1 is associated with glycocalyx and is detached from the cell surface by heparinase or PI-PLC treatment. (**A**,**B**) Binding of Del-1 (50 nM) to immobilized BSA, HS (50 nM), and SDC1 (50 nM) and binding of Del-1 to immobilized HS or SDC1 upon heparinase treatment (0.005 U/ml or 0.05 U/ml), as assessed by solid-phase binding assay. The data are shown as the mean ± SD (n = 3 mice per group). ^*^P < 0.05 and ^†^*P* < 0.01, Student’s *t*-test. (**C**) Immunohis*t*ochemistry for Del-1 treated with heparinase (0.05 U/ml) or PI-PLC (2 U/ml) at 37 °C for 1.5 h. Nuclei were stained with DAPI (blue). Scale bars, 50 μm. (**D**) ELISA for Del-1 from the supernatants from the culture treated with heparinase (0.5, 2 U/ml) or PI-PLC (0.2, 2 U/ml) at 37 °C for 1 h. The data are shown as the mean ± SD (n = 6 mice per group). ^*^P < 0.05 and ^†^*P* < 0.01, Student’s *t*-test. BSA: bovine serum albumin; DAPI: 4′,6-diamidino-2-phenylindole; Del-1: developmental endothelial locus-1; ELISA: enzyme-linked immunosorbent assay; HS: heparan sulfate; PI-PLC: phosphatidylinositol-specific phospholipase C; SDC1: syndecan-1.

**Figure 2:**
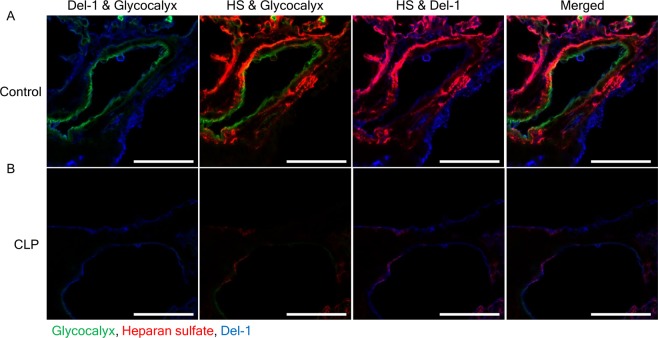
Sections of lung from CLP and control mice stained for heparan sulfate, Del-1, and glycocalyx. Scale bars, 50 μm. (**A**) The endothelial glycocalyx was mostly intact, and heparan sulfate and Del-1 were spatially distributed in the endothelial cell and extracellular matrix. (**B**) After 24 h of CLP, the endothelial glycocalyx was almost completely degraded with less formation of heparan sulfate and Del-1. CLP: cecal ligation and puncture; Del-1: developmental endothelial locus-1; HS: heparan sulfate.

The lung tissue from wild-type (WT) mice displayed abundant Del-1 staining. As expected, no Del-1 staining was detected in Del-1^−/−^ tissue (see Supplementary Fig. S2). The relatively restricted pattern of glycocalyx staining in the surface layer of the pulmonary vascular endothelium is contrasted with Del-1 staining involving the endothelial surface layer, an endothelial cell, and the extracellular matrix (see Supplementary Fig. S3). We investigated the ability of Del-1 to interact with heparan sulfate and syndecan-1, which is a major heparan sulfate proteoglycan constituting endothelial glycocalyx. Del-1 bound to heparan sulfate and syndecan-1 (Fig. 1A,B). Importantly, this Del-1 binding was largely dependent on heparinase; pretreatment of the cells with the enzyme strongly reduced Del-1 binding (Fig. 1A,B). Moreover, immunohistochemistry showed that Del-1 was detached from the cell surface by heparinase or PI-PLC treatment (Fig. 1C). Del-1 level was also measured by ELISA in the supernatant of HEK293T cells expressing mouse Del-1 after heparinase or PI-PLC treatment. This increased Del-1 levels significantly in the supernatant compared to control cells (Fig. 1D). These findings suggest that Del-1 is linked with the endothelium. In the lung sections from the control mice, a mostly intact glycocalyx and spatial distribution of heparan sulfate and Del-1 were verified by immunostaining (Fig. 2A). Lower levels of glycocalyx, heparan sulfate, and Del-1 staining were detected in the CLP mice (Fig. 2B).

### Increased inflammatory cell recruitment *in vivo* due to Del-1 deficiency

**Figure 3:**
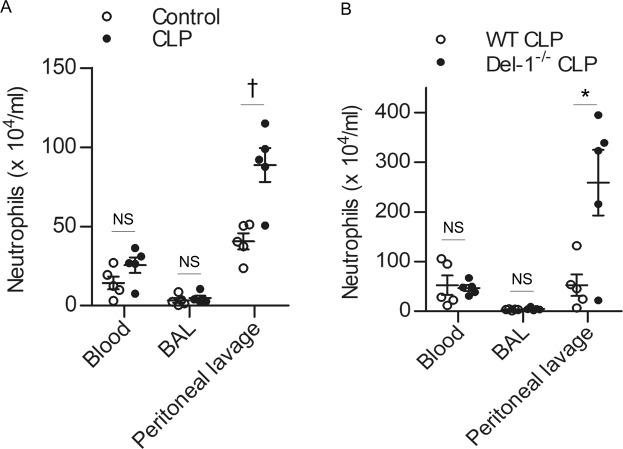
Del-1 deficiency increases inflammatory cell recruitment. (**A**) The numbers of neutrophils in the blood, BAL fluid, and peritoneal lavage fluid in control and CLP mice at 4 h after CLP. (**B**) The numbers of neutrophils in the blood, BAL fluid, and peritoneal lavage fluid in WT and Del-1^−/−^ mice at 4 h after CLP. The data are shown as the mean ± SD (n = 5 mice per group). ^*^P < 0.05 and ^†^*P* < 0.01, Student’s *t*-test. BAL: bronchoalveolar lavage; CLP: cecal ligation and puncture; Del-1: developmental endothelial locus-1; WT: wild-type.

As expected, CLP mice displayed significantly higher accumulation of neutrophils in the peritoneal lavage fluid than did control mice (Fig. 3A). In addition, Del-1^−/−^ CLP mice demonstrated significantly higher accumulation of neutrophils in the peritoneal lavage fluid than did WT CLP mice (Fig. 3B). The increased leukocyte recruitment in Del-1^−/−^ CLP mice could not be attributed to an alteration in peripheral blood counts, because constitutive leukocyte numbers in the blood were comparable between WT CLP and Del-1^−/−^ CLP mice (Fig. 3B).

### Del-1 levels in various tissues in the septic animal model

**Figure 4:**
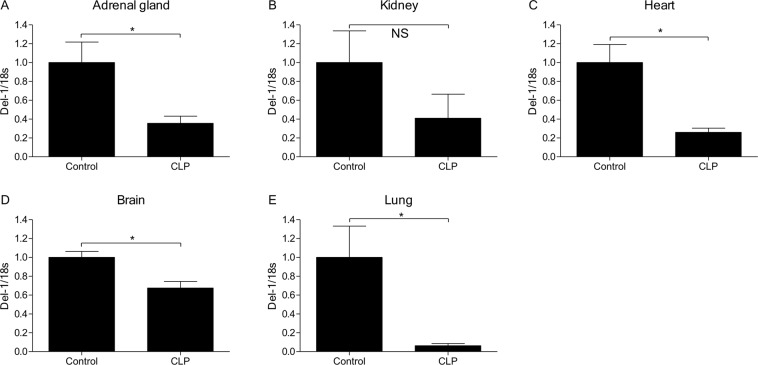
RT-PCR quantification of Del-1 mRNA expression in various mouse tissues during sepsis. After 24 h of CLP, relative quantification of Del-1 expression shows that CLP significantly decreased Del-1 mRNA expression in adrenal gland, heart, brain, and lung tissue, but not kidney. The data are shown as a ratio of the control, which is set as 1.0. The data are shown as the mean ± SEM (n = 5 mice per experimental group and n = 3 mice per control group). ^*^P < 0.05, Mann-Whitney *U* test (Student’s *t*-test in **D**). CLP: cecal ligation and puncture; Del-1: developmental endothelial locus-1; RT-PCR: real-time polymerase chain reaction.

After 24 h of CLP, expression of Del-1 was significantly decreased in the adrenal gland, heart, brain, and lung tissues, but not kidney (Fig. 4).

### Serum and BAL fluid Del-1 levels in the septic animal model

**Figure 5:**
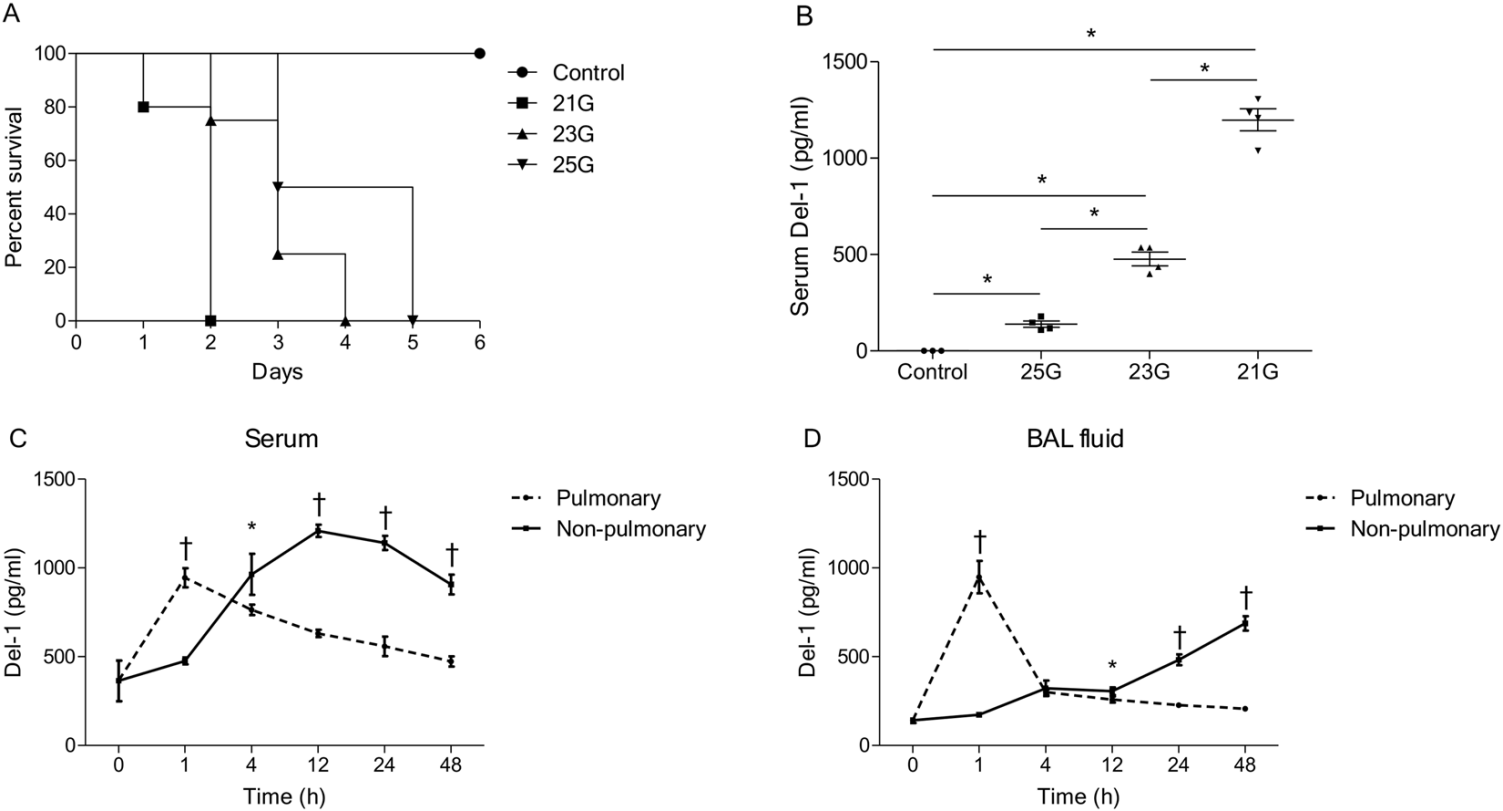
Del-1 levels in the serum and BAL fluid from a septic animal model. (**A**) The CLP mice with less severe sepsis (25-gauge needle puncture size) died within 5 days whereas those with more severe sepsis (21-gauge needle puncture size) died within 2 days (n = 4–5 mice per experimental group and n = 3 mice per control group; *P* = 0.01, log-rank test, compared by Kaplan-Meier estimates). (**B**) After 24 h of CLP, serum Del-1 levels were significantly increased in accordance with the severity of sepsis. The data are shown as the mean ± SEM (n = 4–5 mice per experimental group and n = 3 mice per control group). ^*^P < 0.01, Student’s *t*-test. (**C**) The serum Del-1 levels peaked 1 h after instillation of LPS in the pulmonary sepsis group (dashed line). In the non-pulmonary sepsis group, serum Del-1 levels were high at 12 h after CLP (21-guage, solid line). (**D**) Del-1 levels in BAL fluid peaked 1 h after instillation of LPS. In the CLP (21-guage) mice, BAL fluid Del-1 was high at 48 h after procedure. The data are shown as the mean ± SD (n = 6 mice per group). ^*^P < 0.05 and ^†^*P* < 0.01, pulmonary sepsis vs. non-pulmonary sepsis, Student’s *t*-test. BAL: bronchoalveolar lavage; CLP: cecal ligation and puncture; Del-1: developmental endothelial locus-1; LPS: lipopolysaccharide.

Mice that underwent CLP with a 25-gauge needle (less severe sepsis) died within 5 days of surgery and those that underwent CLP with a 21-gauge needle (more severe sepsis) died within 2 days of surgery (Fig. 5A). Serum Del-1 levels were significantly higher in the CLP mice than in the control mice and were significantly increased with increasing severity of sepsis (Fig. 5B).

In the mice with LPS-induced lung inflammation (pulmonary sepsis), the serum Del-1 levels peaked 1 h after administration of LPS (Fig. 5C). In the CLP mice (non-pulmonary sepsis), serum Del-1 levels were high at 12 h after procedure (Fig. 5C). We also evaluated concentration of Del-1 in BAL fluid over time in both models. Similar to serum Del-1, Del-1 levels in BAL fluid peaked 1 h after administration of LPS (Fig. 5D). Meanwhile, BAL fluid Del-1 in the CLP mice was high at 48 h after procedure (Fig. 5D).

### Other serum biomarker levels in pulmonary and non-pulmonary sepsis

**Figure 6:**
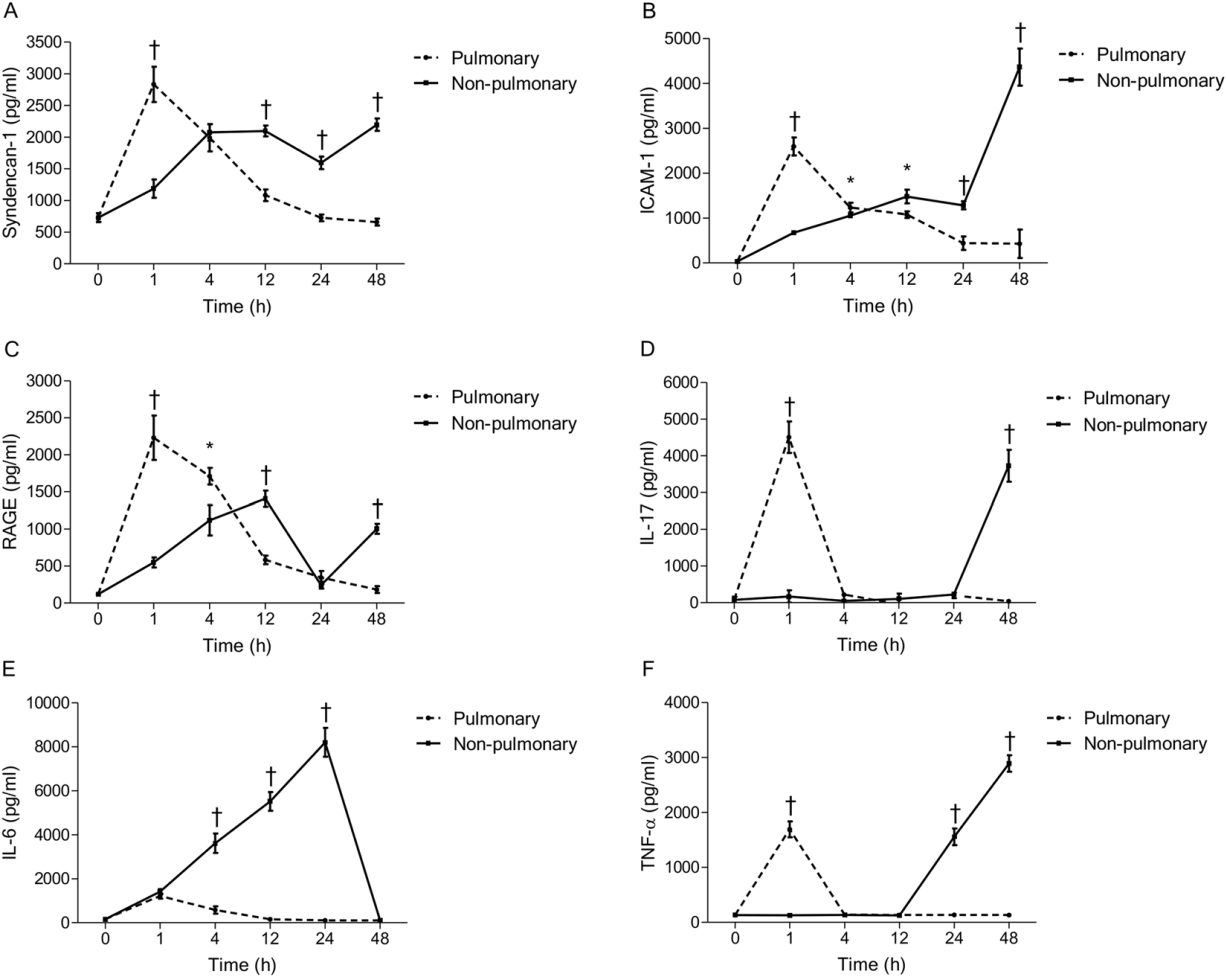
Biomarker levels in pulmonary and non-pulmonary sepsis models. (**A**–**C**) Serum levels of syndecan-1, ICAM-1, and RAGE all peaked 1 h after LPS instillation in the pulmonary sepsis group. In the non-pulmonary sepsis group, levels of these serum biomarkers after CLP peaked at 4 h–12 h for syndecan-1, 48 h for ICAM-1, and 12 h for RAGE. (**D**–**F**) These findings were largely similar for inflammatory biomarkers (IL-17, IL-6, and TNF-α). The data are shown as the mean ± SD (n = 6 mice per group). ^*^P < 0.05 and ^†^*P* < 0.01, pulmonary sepsis vs. non-pulmonary sepsis, Student’s *t*-test. CLP: cecal ligation and puncture; ICAM: intercellular cell adhesion molecule; IL: interleukin; LPS: lipopolysaccharide; RAGE: receptor for advanced glycation end products; TNF: tumor necrosis factor.

In the pulmonary sepsis model, LPS-induced serum levels of syndecan-1, ICAM-1, RAGE, IL-17, IL-6, and TNF-α all peaked 1 h after LPS administration (Fig. 4). In the non-pulmonary sepsis model, levels of these serum biomarkers after CLP peaked at 4 h–12 h for syndecan-1, 48 h for ICAM-1, 12 h for RAGE, 48 h for IL-17, 24 h for IL-6, and 48 h for TNF-α (Fig. 6).

### Patient characteristics

**Figure 7:**
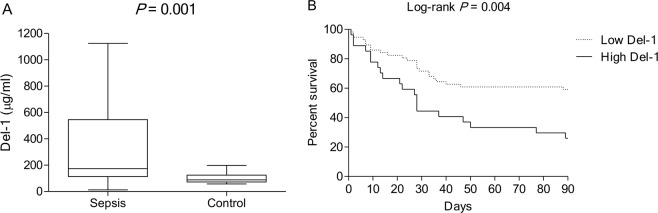
Serum Del-1 levels in the human samples. (**A**) Comparison of serum Del-1 levels in patients with sepsis and those in the controls. The boxplots show the median with the 25^th^ and 75^th^ percentiles. The whiskers show the 5^th^ and 95^th^ percentiles (n = 84 in the sepsis group and n = 20 in the control group). The *P*-value indicates the result of the Mann-Whitney *U* test. (**B**) Kaplan-Meier survival curves for the low Del-1 and high Del-1 groups. Del-1: developmental endothelial locus-1.

The median serum Del-1 levels in the 84 patients and 20 healthy control subjects were 174.0 (range, 113.7–534.4) µg/ml and 88.2 (range, 73.5–120.5) µg/ml, respectively (*P* = 0.001; Fig. 7A). The optimal cutoff serum Del-1 level that predicted 90-day mortality in the patients was 375.96 µg/ml.

The patients were divided into a low Del-1 group (serum Del-1 <375.96 µg/ml; n = 57) and a high Del-1 group (serum Del-1 ≥375.96 µg/ml; n = 27). The baseline characteristics and clinical outcomes in these two groups are shown in Table 1. The groups were similar in terms of age, sex, and Charlson Comorbidity Index. The proportion of patients with non-pulmonary sepsis tended to be higher in the high Del-1 group (23% vs. 41%; *P* = 0.09). The high Del-1 group had a significantly higher SOFA score (10 [range, 7–12] vs. 12 [range, 10–14]; *P* = 0.02) and DIC score (3 [range, 2–4] vs. 4 [range, 3–6]; *P* = 0.02) when admitted to the ICU. Likewise, there were trends towards a higher APACHE II score, a higher proportion of patients with septic shock, and a higher proportion of patients receiving mechanical ventilation in the high Del-1 group. The high Del-1 group had a significantly lower platelet count and prothrombin time (%). Initial serum lactate was also higher in the high Del-1 group, but the difference was not statistically significant (*P* = 0.08).

**Table 1:** Baseline characteristics and clinical outcomes in the low Del-1 and high Del-1 groups.

Variable	Low Del-1 group (n = 57)	High Del-1 group (n = 27)	*P*-value
**Baseline characteristics**			
Age, years	70 (62–76)	65 (54–74)	0.29
Male sex	35 (61)	19 (70)	0.42
Charlson Comorbidity Index	3 (1–4)	3 (2–6)	0.21
**Source of sepsis**			
Pulmonary	41 (72)	12 (44)	0.02
Non-pulmonary	13 (23)	11 (41)	0.09
Bacteremia	15 (26)	10 (37)	0.32
Septic shock on admission to ICU	44 (77)	24 (89)	0.20
ARDS on admission to ICU	9 (16)	4 (15)	>0.99
APACHE II score on admission to ICU	23 (18–27)	25 (21–29)	0.26
SOFA score on admission to ICU	10 (7–12)	12 (10–14)	0.02
DIC score on admission to ICU	3 (2–4)	4 (3–6)	0.02
Overt DIC^*^	12 (21)	11 (41)	0.059
Use of mechanical ventilation in day 1	34 (60)	20 (74)	0.20
Use of a vasopressor on day 1	38 (67)	21 (78)	0.30
**Laboratory data on day 1**			
White cell count, 1000/mm^3^	14.8 (10.7–21.2)	13.3 (5.4–18.8)	0.13
Platelet count, 1000/mm^3^	134 (70–243)	93 (49–136)	0.02
Prothrombin time, %	64 (55–80)	43 (37–62)	<0.001
C-reactive protein, mg/dl	18.8 (6.8–26.2)	10.8 (6.3–20.0)	0.10
Procalcitonin, ng/ml	4.4 (0.6–26.2)	5.4 (0.7–19.5)	0.96
Lactate, mmol/l	2.5 (1.2–3.9)	4.0 (1.7–7.3)	0.08
**Outcomes**			
Length of stay, d			
ICU	4 (2–16)	5 (2–12)	0.77
Hospital	21 (10–34)	32 (14–55)	0.07
**Mortality**			
28-day	12 (21)	11 (41)	0.059
90-day	23 (40)	20 (74)	0.004

The data are presented as the median (interquartile range) or number (percentage) of patients. The *P*-values indicate the results of comparing the low Del-1 and high Del-1 groups using the Mann-Whitney *U*, chi-square, or Fisher’s exact test. ^*^A DIC score of five or more. APACHE: Acute Physiology and Chronic Health Evaluation; ARDS: acute respiratory distress syndrome; Del-1: developmental endothelial locus-1; DIC: disseminated intravascular coagulation; ICU: intensive care unit; SOFA: Sequential Organ Failure Assessment.

### Clinical outcomes in the low Del-1 and high Del-1 groups

There was no difference in the resuscitation or infection goal achieved between the groups (see Supplementary Table S1); however, the 90-day mortality rate was significantly higher in the high Del-1 group than in the low Del-1 group (40% vs. 74%; *P* = 0.004; Table 1). Moreover, there were trends towards a higher 28-day mortality rate and a longer hospital stay in the high Del-1 group (Table 1). Subgroup analysis that did not include pulmonary sepsis showed a significant difference in the 90-day mortality rate in favor of the high Del-1 group (see Supplementary Table S2). The Kaplan-Meier survival curves for the low and high Del-1 groups are shown in Fig. 7B (*P* = 0.004).

### Association between the serum Del-1 level and mortality

Multivariate analysis revealed a significant association of a low platelet count with higher 90-day mortality. A high serum Del-1 level tended to be associated with higher mortality (adjusted odds ratio, 1.87; 95% CI, 0.995–3.50; *P* = 0.052; see Supplementary Table S3); subgroup analysis showed that this relationship was only statistically significant in the non-pulmonary sepsis group (see Supplementary Table S4). The AUCs of the serum Del-1 for predicting 28-day and 90-day mortality were 0.63 (95% CI, 0.49–0.76) and 0.65 (95% CI, 0.53–0.77), respectively. In the non-pulmonary sepsis group, however, the AUCs of the serum Del-1 for predicting 28-day and 90-day mortality were 0.84 (95% CI, 0.62–1.00) and 0.70 (95% CI, 0.47–0.93), respectively. These were larger than the AUCs for the APAHCE II and SOFA scores (see Supplementary Table S5).

### Inflammatory cytokines and serum Del-1

The IL-6 level correlated weakly with the serum Del-1 level (see Supplementary Table S6). The serum IL-6 level was significantly higher in the high Del-1 group than in the low Del-1 group (see Supplementary Table S7).

## Discussion

The main findings of the present study are as follows. First, shedding of the endothelial glycocalyx and washout of Del-1 were observed in an animal model of sepsis. Second, serum Del-1 increased in the animal model according to the severity of sepsis, and expression profiles of serum Del-1 over time between non-pulmonary and pulmonary sepsis models were different. Third, serum Del-1 was higher in septic patients than in controls, and high serum Del-1 was associated with more severe sepsis and higher mortality. This study expands the findings of previous reports suggesting that Del-1 plays an important role in both acute and chronic inflammation^8,10,24–26^.

Several pathophysiologic processes have been associated with structural and functional derangement of the glycocalyx^27,28^. The strong association between degradation of the glycocalyx and disease may also exist for sepsis^29,30^. The present study demonstrated that together with membrane-bound molecules such as glycocalyx and heparan sulfate, Del-1 forms the endothelium. In this study, endothelium-derived Del-1 was shown to inhibit leukocyte-endothelial interactions in an animal model of sepsis. In the septic condition, the expression of endothelial cell specific molecules is less evident, suggesting shedding of the glycocalyx. Moreover, serum Del-1 increased with increasing severity of sepsis and was associated with the prognosis. These findings support the notion that endothelial dysfunction has a prominent role in the pathophysiology of sepsis.

The inflammatory response contributes to life-threatening multiorgan dysfunction in the course of sepsis^5^. Several studies have demonstrated that pro-inflammatory stimuli, such as LPS, TNF-α, and IL-17, lead to increased expression of inflammation-promoting adhesion receptors and to reduced expression of inflammation-inhibiting signals such as Del-1^8–10,24,26,31,32^. In this study, there was a decrease in Del-1 expression in various tissues in septic mice when compared to control mice. This finding is consistent with previous reports that expression of Del-1 in the tissues is affected by the course of inflammation^8,10,24–26,31,33^. Interestingly, Del-1 expression decreased in the adrenal gland tissue from septic mice. Pro-inflammatory cytokine-induced adrenal Del-1 deficiency may promote accumulation of leukocytes in the adrenal gland and subsequently affect adrenal function in the course of sepsis^24^. This phenomenon may have contributed to the increased mortality in our patients with high serum Del-1 as the presence of adrenal dysfunction in septic shock has a high mortality rate^34,35^. Unfortunately, we did not have data on serum and urinary albumin/creatinine or serum corticosterone and ACTH concentrations that may be useful to identify renal or adrenal dysfunction in an animal model of sepsis and in humans. Thus, confirmatory studies are required.

Although there are several reports on expression of Del-1 in body tissues^8,10,24–26^, there are no reports on Del-1 levels in blood. The issues concerning tissue biomarkers include the problems of obtaining repeated samples and their cost. Unlike other tissues, blood samples can be easily obtained. We demonstrated that disruption and shedding of the glycocalyx during sepsis may liberate Del-1 from endothelial cells and the extracellular matrix, allowing it to enter into the bloodstream and resulting in an increased serum Del-1 level. This finding is concordant with previous observations that serum levels of syndecan-1 and circulating glycosaminoglycans are increased in patients with critical illness^14,30,36^. Further investigations in this regard are warranted.

To further translate the current results from animals to humans, blood samples were obtained from critically ill patients with sepsis and from healthy controls. Serum Del-1 was significantly higher in the patients with sepsis than in the control subjects. Moreover, high serum Del-1 was associated with more severe disease, organ dysfunction, and mortality. Interestingly, a higher DIC score, a lower platelet count, and a lower prothrombin time (%) were observed in patients with high serum Del-1. Formation of microparticles accompanied by externalization of phosphatidylserine has an essential procoagulant role^37^ and Del-1 participates in the clearance of phosphatidylserine-expressing microparticles^38^. Therefore, endothelial damage causing Del-1 depletion (indicated by a high Del-1 level in blood) may contribute to the procoagulant state, with pathologically increased generation of microparticles. Leukocyte-platelet aggregates is accompanied by increased expression of inflammatory genes and procoagulant effects^39^. Del-1 also inhibits formation of leukocyte-platelet aggregates by blocking the interaction between Mac-1 and glycoprotein Ib^40^. Finally, shedding of the glycocalyx alone causes coagulopathy in patients with severe trauma and hemorrhagic shock^41^. Overall, these findings indicate that endothelial shedding causing Del-1 depletion may be associated with sepsis-induced coagulopathy.

The present study showed that both the early increase of serum Del-1 (peak 1 h) after LPS instillation and late increase of serum Del-1 (peak 12–48 h) after CLP might reflect inflammatory response. These findings were largely similar for biomarkers of endothelial injury and pro-inflammatory cytokines. The hypertriglyceridemic effect of LPS occurs as rapidly as 2 h after administration^42^. Meanwhile, in a rat model of sepsis, several genes involved in lipid metabolism are upregulated 6 h after CLP^43^. In this study, the proportion of non-pulmonary sepsis was higher in patients with high Del-1, and the association between serum Del-1 level and mortality became more pronounced in non-pulmonary sepsis. In addition, subgroup analysis showed that serum Del-1 was able to predict mortality in non-pulmonary sepsis. Although the samples were collected at ICU admission, it is unlikely that the serum Del-1 level was analyzed few hours after sepsis onset, suggesting that our results more reflect the relationship of serum Del-1 with non-pulmonary sepsis than pulmonary sepsis.

Sepsis is diagnosed based on clinical criteria and does not reflect its pathophysiology^1^. Therefore, sepsis may be very heterogeneous with different responses to therapy. For instance, Famous *et al*. identified ARDS patients responding variously to fluid management^44^. Our findings demonstrate further evidence of sepsis-induced endothelial dysfunction. Serum Del-1 levels may identify distinct subphenotypes of sepsis for therapy targeted to the glycocalyx. Several studies in hemorrhagic shock or ischemia-reperfusion injury have shown that administration of fresh frozen plasma, sevoflurane, or hydrocortisone reduces shedding of the glycocalyx^45–47^. Soluble Del-1 has potential as a treatment for neutrophil-mediated inflammatory diseases^8,10,26,40^. Moreover, a recent study identified Del-1 as a factor promoting myelopoiesis at steady-state and especially under inflammatory conditions^48,49^. This may be important, because neutropenia is a major predictor of outcomes in patients with sepsis. Del-1 may be useful for the diagnosis and treatment of sepsis but requires further evaluation.

Several limitations must be considered when interpreting this study. First, although the relationship of high serum Del-1 and severity of sepsis was robust, it does not fully elucidate the mechanisms via which endothelial dysfunction leads to increased serum Del-1. For instance, Del-1 could have been released from the adrenal gland during sepsis. However, it is plausible that Del-1 sheds from the endothelial glycocalyx. Our findings that Del-1 interacted with endothelial proteoglycan and was detached from the cell surface by heparinase or PI-PLC treatment support this hypothesis. Further studies are needed to determine the source of serum Del-1. Second, intratracheal instillation of LPS causes global lung inflammation and injury, but it may not necessarily represent a “pulmonary sepsis” model. Third, the human study had a retrospective design, which increases the risk of selection bias, and the relatively small sample size of the study is likely to be responsible for some of the non-significant results. Third, the cutoff value of 375.96 µg/ml is somewhat arbitrary.

In summary, we conclude that serum Del-1 could be a useful biomarker of endothelial dysfunction, sepsis, and sepsis-induced organ dysfunction.

## Supplementary information

Supplementary Information

## Data Availability

All data generated or analyzed during this study are included in this published article (and its Supplementary Information files).
