# Supplementary material for: Serum developmental endothelial locus-1 is associated with severity of sepsis in animals and humans
Source: Sci Rep. 2019 Sep 10;9:13005. doi: 10.1038/s41598-019-49564-5 (PMC6737092; doi:10.1038/s41598-019-49564-5)
Supplement: Supplementary file 1 — Supplementary Information [file 41598_2019_49564_MOESM1_ESM.pdf]

Supplementary Information

Serum developmental endothelial locus-1 is associated with severity of sepsis in animals and humans

Won-Young Kim, Seung-Hwan Lee, Dong-Young Kim, Hyun Jin Ryu, Gyu Rak Chon, Yun Young Park, Yan Fu, Jin Won Huh, Chae-Man Lim, Younsuck Koh, Eun Young Choi, and Sang-Bum Hong

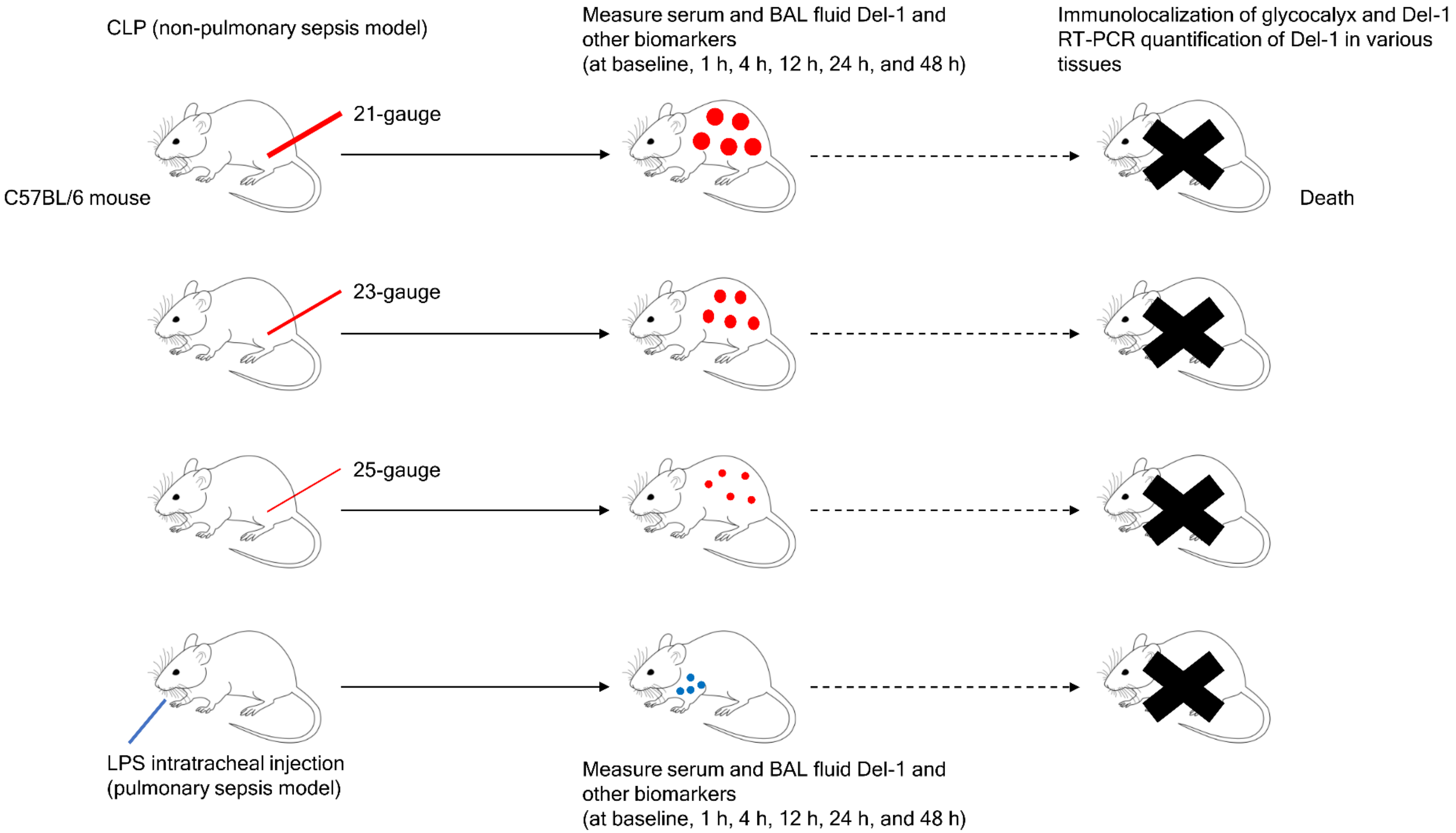

Supplementary Figure S1. Schematic flow chart of the experimental design. BAL: bronchoalveolar lavage; CLP: cecal ligation and puncture; Del-1: developmental endothelial locus-1; LPS: lipopolysaccharide; RT-PCR: real-time polymerase chain reaction.

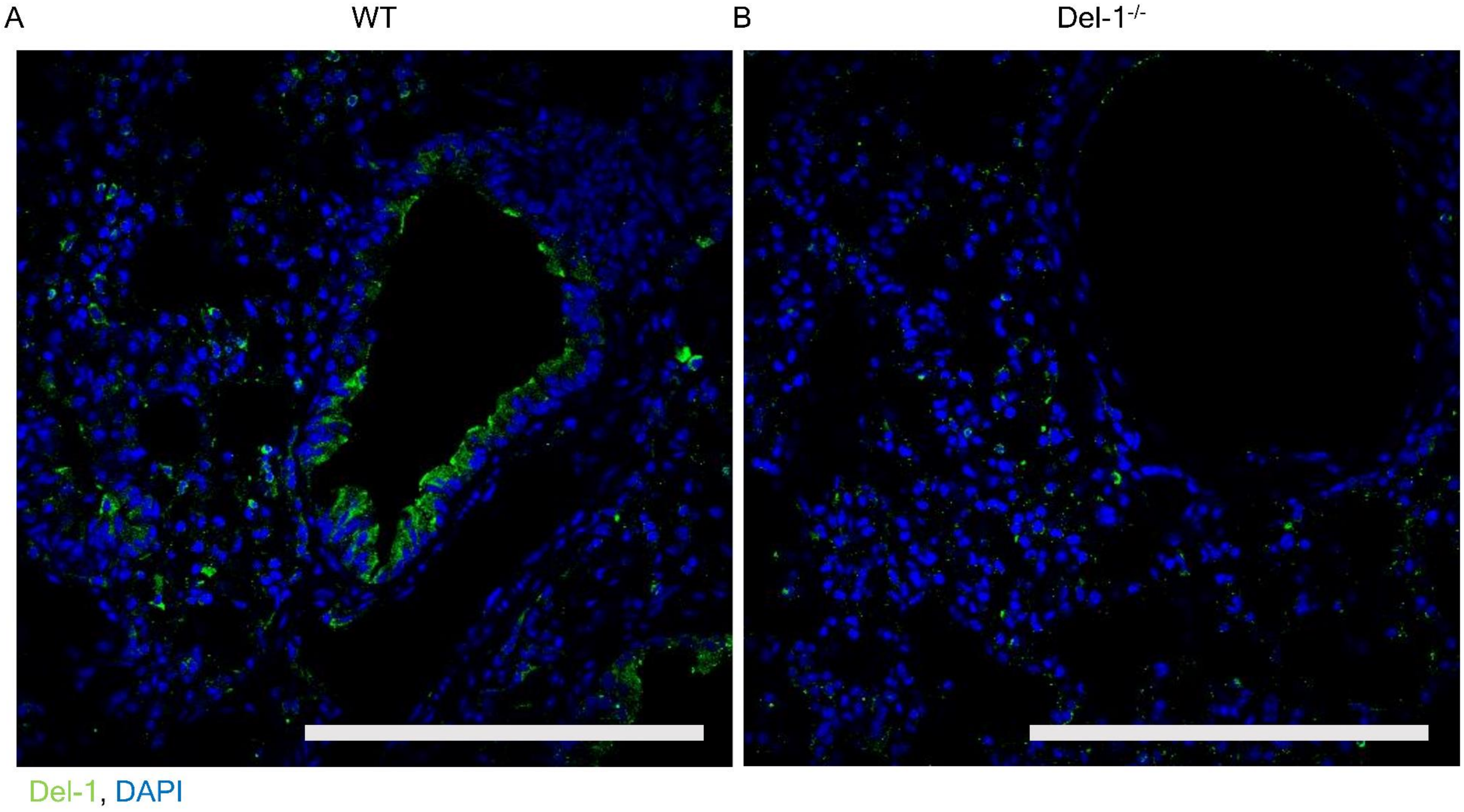

Supplementary Figure S2. Fluorescent confocal images of bronchiolar epithelium from 6–8-week-old WT and Del-1$^{-/-}$ mice stained for Del-1. Scale bars, 200 μm. (**A**) Expression of Del-1 was prominent in the WT bronchioles. (**B**) No positive Del-1 staining was detected in Del-1$^{-/-}$ bronchioles. DAPI: 4',6-diamidino-2-phenylindole; Del-1: developmental endothelial locus-1; WT: wild-type.

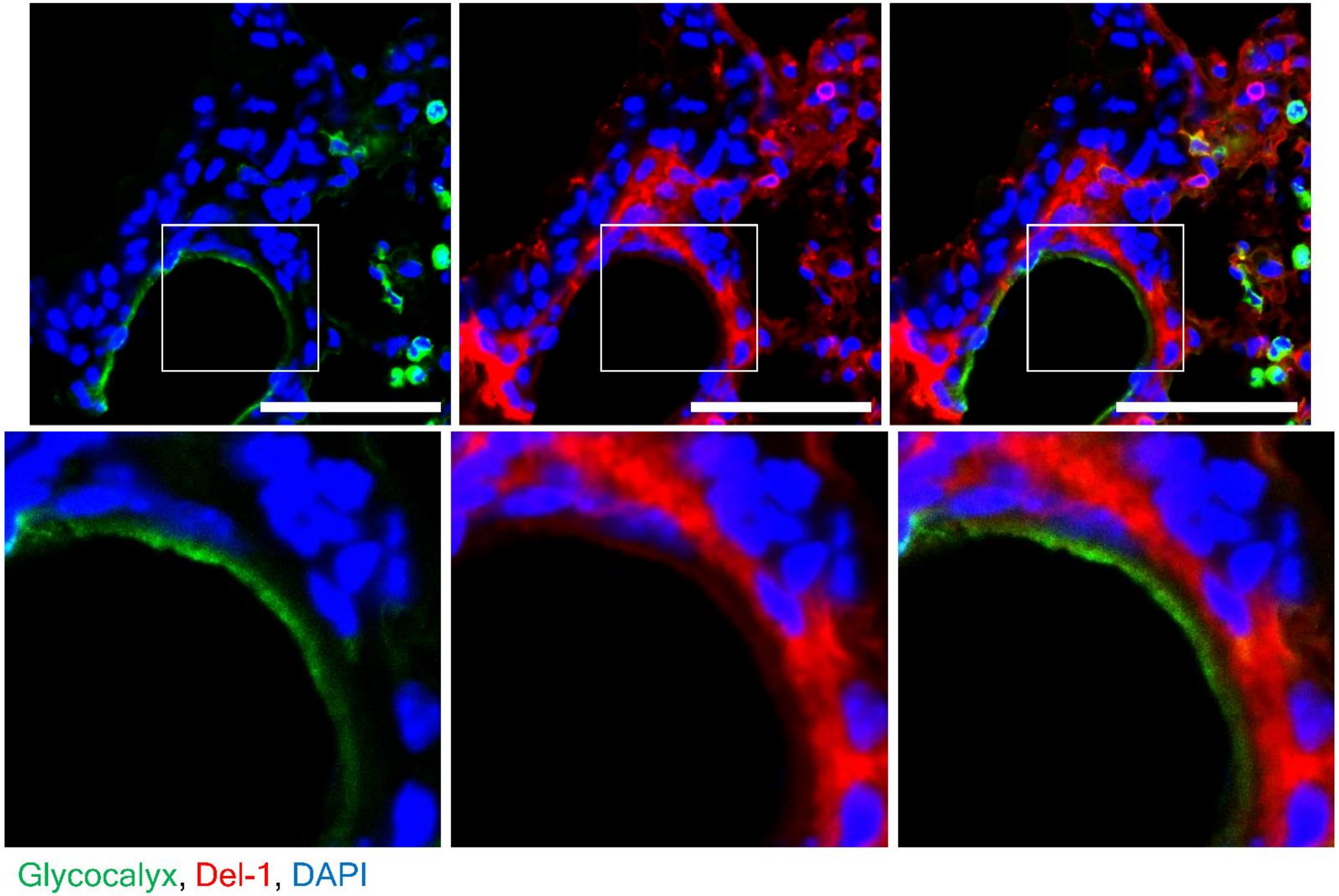

Supplementary Figure S3. Sections of pulmonary vascular endothelium from WT mice stained for glycocalyx (left) or Del-1 (middle); right, merged images. Bottom row, ×630 enlargement of areas outlined above. Expression in the glycocalyx was localized to the endothelial surface layer, although Del-1 staining in the confocal image involved the endothelial surface layer, the endothelial cell, and the extracellular matrix. DAPI: 4',6-diamidino-2-phenylindole; Del-1: developmental endothelial locus-1; WT: wild-type.

Supplementary Table S1. Resuscitation and infection goals achieved.

Variable	Low Del-1 group (n = 57)	High Del-1 group (n = 27)	*P*-value
Central venous pressure ≥ 8 mmHg	39/49 (80)	16/22 (73)	0.55
Mean arterial pressure ≥ 65 mmHg	43/49 (88)	22/24 (92)	>0.99
Central venous oxygen saturation ≥ 70%	17/42 (41)	8/20 (40)	0.97
Adequate antimicrobial therapy*	47 (83)	22 (82)	>0.99

The data are presented as the number (percentage) of patients. *P*-values indicate the results of comparing the low and high Del-1 groups by chi-square or Fisher's exact test. *Defined as when the regimen included one or more antimicrobial agents to which the causative pathogen was susceptible *in vitro*. Del-1: developmental endothelial locus-1.

Supplementary Table S2. Clinical outcomes in the low and high Del-1 groups that did not include pulmonary sepsis.

Variable	Low Del-1 group (n = 16)	High Del-1 group (n = 15)	*P*-value
Length of stay, d			
ICU	2 (1–6)	3 (2–8)	0.24
Hospital	19 (11–44)	34 (16–50)	0.28
Mortality			
28-day	1 (6)	5 (33)	0.08
90-day	3 (19)	10 (67)	0.007

The data are presented as the median (interquartile range) or number (percentage) of patients. *P*-values indicate the results of comparing the low and high Del-1 groups using the Mann-Whitney *U*, chi-square, or Fisher's exact test. Del-1: developmental endothelial locus-1; ICU: intensive care unit.

Supplementary Table S3. Cox regression model with 90-day mortality as the outcome.

Variable	Adjusted HR (95% CI)	*P*-value
Platelet count	0.996 (0.99–1.00)	0.04
High Del-1	1.87 (0.995–3.50)	0.052

Multivariate analyses were adjusted for the Charlson Comorbidity Index, APACHE II score, SOFA score, and whether or not coagulopathy was present on admission to the ICU, use of mechanical ventilation within the first 24 h, platelet count and prothrombin time on day 1, length of ICU stay, and a high Del-1 level. APACHE: Acute Physiology and Chronic Health Evaluation; Del-1: developmental endothelial locus-1; HR: hazard ratio; ICU: intensive care unit; SOFA: Sequential Organ Failure Assessment.

Supplementary Table S4. Cox regression model with 90-day mortality as the outcome in the low and high Del-1 groups that did not include pulmonary sepsis.

Variable	Adjusted HR (95% CI)	*P*-value
Platelet count	0.99 (0.97–0.999)	0.03
High Del-1	4.37 (1.19–16.00)	0.03

Multivariate analyses were adjusted for the platelet count on day 1 and a high Del-1 level. Del-1: developmental endothelial locus-1; HR: hazard ratio.

Supplementary Table S5. Areas under the receiver-operating characteristic curves (AUCs) of the serum Del-1, severity scores, and laboratory data for predicting mortality.

	28-day mortality		90-day mortality	
Variable	AUC	95% CI	AUC	95% CI
All patients (n = 84)				
Del-1	0.63	0.49–0.76	0.65	0.53–0.77
APACHE II score	0.71	0.58–0.83	0.65	0.53–0.76
SOFA score	0.65	0.52–0.78	0.68	0.57–0.80
White cell count	0.44	0.30–0.59	0.39	0.27–0.51
C-reactive protein	0.57	0.44–0.70	0.49	0.37–0.62
Procalcitonin	0.54	0.41–0.68	0.49	0.36–0.61
Lactate	0.69	0.57–0.81	0.62	0.50–0.74
Pulmonary sepsis (n = 53)				
Del-1	0.64	0.48–0.79	0.66	0.51–0.80
APACHE II score	0.69	0.53–0.85	0.71	0.56–0.85
SOFA score	0.65	0.49–0.81	0.68	0.53–0.83
White cell count	0.48	0.32–0.65	0.43	0.27–0.58
C-reactive protein	0.66	0.51–0.81	0.58	0.42–0.74
Procalcitonin	0.61	0.44–0.77	0.53	0.37–0.69
Lactate	0.78	0.65–0.91	0.72	0.58–0.86
Non-pulmonary sepsis (n = 24)				
Del-1	0.84	0.62–1.00	0.70	0.47–0.93
APACHE II score	0.78	0.59–0.96	0.44	0.20–0.68
SOFA score	0.82	0.63–1.00	0.71	0.48–0.93
White cell count	0.63	0.24–1.00	0.49	0.22–0.75
C-reactive protein	0.34	0.03–0.65	0.36	0.12–0.59
Procalcitonin	0.53	0.31–0.74	0.52	0.28–0.76
Lactate	0.71	0.48–0.94	0.62	0.38–0.86

APACHE: Acute Physiology and Chronic Health Evaluation; Del-1: developmental endothelial locus-1; SOFA: Sequential Organ Failure Assessment.

Supplementary Table S6. Correlation between serum Del-1 and inflammatory cytokines.

Variable		IL-1β	TNF-α	IL-6
Del-1	γ	-0.07	0.11	0.24
	P-value	0.52	0.31	0.03

Del-1: developmental endothelial locus-1; IL: interleukin; TNF: tumor necrosis factor.

Supplementary Table S7. Inflammatory cytokine levels in the low and high Del-1 groups.

Variable	Low Del-1 group (n = 57)	High Del-1 group (n = 27)	*P*-value
IL-1β, pg/ml	10.1 (3.2–34.5)	6.3 (2.5–26.7)	0.36
TNF-α, pg/ml	17.6 (9.2–54.4)	19.1 (8.8–59.8)	0.70
IL-6, pg/ml	126.2 (41.9–795.3)	360.5 (91.9–3349.6)	0.02

The data are presented as the median (interquartile range). *P*-values indicate the results of comparing the low and high Del-1 groups using the Mann-Whitney *U* test. Del-1: developmental endothelial locus-1; IL: interleukin; TNF: tumor necrosis factor.
